# Synaptic Interactions Between Serotonergic and Dopaminergic Systems in Parkinson’s Disease

**DOI:** 10.2174/011570159X336597241217062042

**Published:** 2025-02-21

**Authors:** Gioia Marino, Federica Campanelli, Giuseppina Natale, Maria De Carluccio, Federica Servillo, Veronica Ghiglieri, Paolo Calabresi

**Affiliations:** 1 Section of Neurology, Department of Neuroscience, Università Cattolica del Sacro Cuore, Rome, 00168, Italy;; 2 Neurology, Fondazione Policlinico Universitario Agostino Gemelli IRCCS, Rome, 00168, Italy;; 3 Unit of Neurology, IRCCS Neuromed, Pozzilli, IS, Italy;; 4 Department of Neurosciences and Neurorehabilitation IRCCS, Raffaele-Roma, Rome, Italy;; 5 Università Telematica San Raffaele, Rome, 00166, Italy

**Keywords:** Serotonin, dopamine, cognitive function, Parkinson’s disease, synaptic plasticity, neurotransmitter

## Abstract

Since both serotonergic and dopaminergic afferents densely innervate many parts of the central nervous system, intact crosstalk between serotonin (5-HT) and dopamine (DA) transmission is essential for regulating synaptic plasticity in the striatum (STR), prefrontal cortex (PFC), and hippocampus (HPC). Experimental models have provided strong evidence of a synergistic action of DA and 5-HT convergent release in PFC, HPC, and STR to modulate motor control, learning, and memory processes. In this review, we will discuss the mechanisms underlying the actions of agonists and antagonists of 5-HT and DA receptors on striatal synaptic plasticity in physiological conditions and Parkinson's disease (PD), a movement disorder in which an imbalance of these two neurotransmitter systems has been hypothesized. This review will also discuss the interactions between 5-HT and DA in PFC and HPC, with particular regard to the influence of this crosstalk on synaptic plasticity and learning. Finally, we will provide an overview of how stimulation or inhibition of DA and 5-HT receptors affects these neurotransmitter expression levels in the three brain regions of interest.

## 1 INTRODUCTION

### 1.1 DA and 5-HT Interactions in the Striatum

**Fig. (1):**
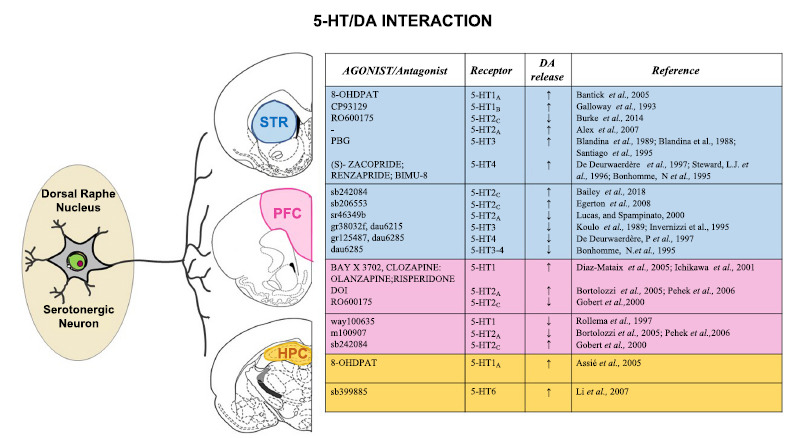
Schematic representation of 5-HT receptors distribution in the striatum, prefrontal cortex and hippocampus. Effects of selective 5-HT receptor agonists/antagonists on dopamine release in STR, PFC, and HC.

Brain monoamine neurotransmitters dopamine (DA) and serotonin (5-HT) play an essential role in modulating the activity of a variety of brain areas within the nervous system (Fig. **1**).

The striatum (STR) is considered an essential structure in the basal ganglia circuit because it receives complex information from cortical and thalamic areas [1, 2]. This region is the largest subcortical structure of the basal ganglia and a critical component of the motor and reward systems that receive both dopaminergic and serotonergic inputs. Dopaminergic and serotonergic neurotransmissions are also present in the regions of the prefrontal cortex (PFC) [3, 4] and the hippocampus (HPC) [2, 5]. These monoaminergic inputs, critical for the modulation of cognitive functions, control neural excitability in different ways. It has been reported that the endogenous 5-HT system influences striatal DA release with the involvement of several 5-HT receptors [6], suggesting that a mechanism based on the interaction between DA and 5-HT systems underlies striatal-dependent processes [7].

Long-term forms of synaptic plasticity, *i.e*., Long-Term Potentiation (LTP) and Long-Term Depression (LTD), are DA-dependent processes physiologically expressed by striatal spiny projection neurons (SPNs) at the level of corticostriatal synapses. Here, they play a central role in shaping the motor learning processes and in the modulation of sensorimotor and visuospatial cognitive functions [8-10].

Corticostriatal LTD is strongly dependent on the action of the neurotransmitter glutamate on α-amino-3-hydroxy-5-methyl-4-isoxazole propionic acid receptors (AMPAR) expressed by SPNs and requires the simultaneous activation of DA D_1_ (D_1_R) and D_2_ (D_2_R) receptors [8] achieved during tonic DA release. Multiple mechanisms, however, regulate this form of plasticity [11].

Glutamatergic N-methyl-D-aspartate channel receptors (NMDAR) are typically involved in the expression of LTP that can be induced when the cell membrane is depolarized in response to a stimulus or when the magnesium ions (Mg^2+^) that bind to the NMDAR channel pore are removed from the external medium. This form of plasticity is preferentially triggered under phasic DA release and requires the selective activation of D_1_R.

Besides glutamatergic and dopaminergic innervations, serotonergic inputs from the dorsal raphe nucleus neurons have been accounted as relevant players that regulate striatal plasticity [12].

Early behavioral, electrophysiological, and biochemical studies have shown that 5-HT may affect striatal DA transmission and thus influence the expression of DA-dependent plasticity. According to these findings, bath application of 5-HT or agonists of 5-HT receptors (5-HTR) alters the dopaminergic system [13-15], and some evidence supports the notion that 5-HT, besides acting as a neurotransmitter, may also function as a growth regulator during specific central nervous system developmental events [16]. In support of this view, due to the strategic distribution of the 5-HTR in the central and peripheral nerve cells [17], a dysregulation of serotonergic signaling during the early stages of brain development negatively affects the formation of neural circuits [18, 19].

#### 1.1.1 5-HT and DA Release

In the dorsal striatum (DS), several subtypes of 5-HTR, either associated with inhibitory or stimulatory G proteins, can be found at both presynaptic and postsynaptic sites (Fig. **1**) [20], further reinforcing the role of the serotonergic system in the striatal synaptic transmission [21] and the concept that 5-HT-DA interaction tunes both excitatory and inhibitory transmissions. The modulatory effects of 5-HT on basal DA release have been described *in vitro* and *in vivo*, more frequently with enhancing [13-15] than with inhibitory effects [6]. Hence, pharmacological manipulations of 5-HTR can help control DA release in the brain areas where the DA/5-HT interaction is possible, such as the STR, the HPC, and the PFC (Fig. **1**).

Serotonin receptors in the STR can promote or prevent the striatal DA neurotransmission.

Activation of 5-HT_2C_R with 5-HT_2C_R agonists, such as Ro600175, reduces DA release [22]. At the same time, antagonism (SB206553 and SB242084) of this receptor favors the release of this neurotransmitter (Fig. **1**) [22-24] while activation of other receptors, such us 5-HT_1A_, 5-HT_1B_, 5-HT_2A_, 5-HT_3_ and 5-HT_4_, leads to its increase (Fig. **1**) [25]. Accordingly, *in vivo*, microdialysis studies have shown that the CP93129, a 5-HT_1B_R agonist, and 8-OH-DPAT, a 5-HT_1A_R agonist, increase DA outflow in the rat STR [26, 27].

The role that these two receptors play in the striatal synapse is made more complex by the fact that both have a presynaptic localization, as autoreceptors, in serotonergic nerve terminals. Still, the 5-HT_1A_R can be found on glutamatergic nerve terminals, and the 5-HT_1B_R are also expressed on SPNs [28].

DA release blockade can also be observed following systemic administration of the antagonist 5-HT_2A_R SR46349B, although this inhibition can be observed under specific conditions. In fact, the application of 5-HT_2A_R antagonists in basal conditions does not change the extracellular concentrations of striatal DA. However, an inhibition of DA release was observed only after amphetamines were administered [29].

The 5-HT_3_R are also present at the striatal level, where they seem to facilitate the release of DA from nigrostriatal neurons, as perfusion with the 5-HT_3_R agonist phenylbiguanide (PBG) leads to a dose-dependent increase in striatal DA [30-32]. Accordingly, 5-HT_3_R antagonists (GR38032F, DAU6215, and DAU 6285) produce a selective reduction of extracellular striatal DA [33, 34].

Concerning the 5-HT_4_R subtype, this group is abundantly distributed in the STR since immunohistochemistry (IHC), and *in situ* hybridization analyses can detect high levels of 5-HT_4_R protein and messenger RNA (mRNA) levels in the DS. Autoradiographic studies show that 5-HT_4_R are heteroreceptors located on the somatodendritic and axon terminals of the spiny efferent neurons within the STR [35], where these receptors are mainly expressed on dorsal and ventral gamma-aminobutyric acid (GABA)-positive neurons [36], but not detectable in the DA terminals [37, 38]. Interestingly, several pieces of evidence demonstrated that stimulation of the 5-HT_4_R leads to increased striatal DA *in vivo*. Studies done on anesthetized rats showed that local perfusion in the vicinity of striatal DA terminals of 5-HT or 5-HT_4_R agonists [[S]-zacopride, renzapride, or BIMU-8 agonists] causes a concentration-dependent increase in striatal DA release [39-41]. This effect can be attenuated following perfusion with selective 5-HT_4_R antagonists (GR125487 and DAU6285) [39].

However, since 5-HT_4_R antagonism does not alter the basal DA efflux, it implies that 5-HT_4_R do not modulate the tonic release of nigrostriatal DA. Still, it rather appears to influence the nigrostriatal DA pathway only after activity-dependent or pharmacological stimulation of the DA and 5-HT systems [25].

#### 1.1.2 5-HT and Striatal Synaptic Plasticity

**Fig. (2):**
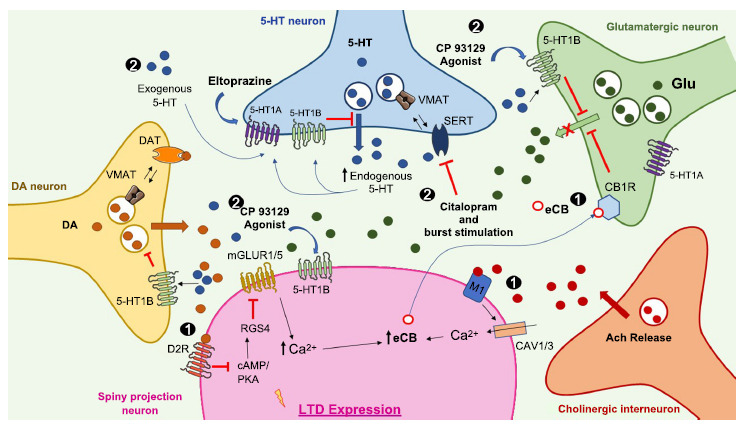
LTD expression depends on the mutually occlusive mechanisms of 5-HT-LTD and endocannabinoids (eCB)-LTD. Activation of 5-HT1b presynaptic receptors with the application of exogenous or endogenous 5-HT leads to a dependent LTD. This mechanism prevents the alternative mechanism process of LTD dependent on eCB from being observed.

Given the importance of DA in regulating corticostriatal synaptic plasticity, several studies investigated whether possible interactions between the serotonergic and dopaminergic systems could influence the direction of the DA-dependent striatal synaptic plasticity (*i.e*., depression *vs.* potentiation). Given the glutamatergic nature of these phenomena, the effect of 5-HT on glutamatergic transmission was also extensively studied in striatal SPNs. In particular, the application of 5-HT, or a burst stimulation coupled with selective 5-HT transporter (SERT) blockade with the selective serotonin reuptake inhibitors (SSRI) citalopram, or with the 5-HT_1B_R agonist CP-93129, was instrumental in understanding the serotonergic modulation of corticostriatal LTD (Mathur *et al*., 2011), with implication for the control of voluntary movements and motor skill learning. This kind of plasticity, described for the first time by Lovinger’s group, was called serotonin-dependent LTD (5-HT-LTD). An application of 5-HT or an increase in endogenous 5-HT is both able to induce an LTD that depends on the activation of presynaptic 5-HT1b receptors. This mechanism occludes the alternative process of endocannabinoids (eCB)/DA-dependent LTD (Fig. **2**) [7]. These data demonstrate that under physiological conditions, activation of D2Rs reduces striatal glutamatergic transmission through a presynaptic mechanism.

This form of 5-HT-LTD is mutually occlusive of other mechanisms that control LTD in the DS. In fact, in striatal SPNs, the activation of D_2_Rs indirectly leads to the activation of (mGLUR_1/5_) and acetylcholine (Ach) release from cholinergic neurons (ChLs). This neurotransmitter binds to muscarinic receptors (M_1_R) located on the SPNs, leading to an increase in Ca^2+^. The increase of Ca^2+^ is responsible for the synthesis of high levels of eCB with a retrograde action on cannabinoid receptors (CB_1_R) located on both cortical and thalamic glutamatergic presynaptic terminals inducing eCB-dependent LTD (eCB-LTD) of glutamatergic synaptic transmission [42-44].

This eCB-LTD mechanism is mutually occlusive with endogenous 5-HT-LTD, suggesting a level of functional competition between the two monoaminergic systems [7].

Results from independent research groups confirmed a unique and mutually occlusive monoaminergic mechanism controlling presynaptic inhibition of corticostriatal glutamate release, further strengthening the concept that important interaction between the DA and 5-HT systems influences striatal-dependent behaviors. This idea is also supported by the observation that neonatal DA depletion in rats leads to serotonergic hyperinnervation in the STR [45, 46].

Further advances in the study have been provided by a study investigating the influence of serotonergic signaling on plasticity at glutamatergic synapses in the D1-expressing subpopulation of SPNs [47]. Using chemogenetic and optogenetic approaches, the authors found that a blockade of serotonergic signaling enables LTD at thalamostriatal synapses. This LTD is achieved as a result of reduced activity of the 5-HT_4_R subtypes, which controls dendritic Ca^2+^ signaling. The results showed for the first time an essential role of 5-HT in the induction of thalamostriatal LTD in SPNs of the direct pathway [47].

 While most of the studies focused on the role of 5-HT on DA-dependent LTD, few addressed the interaction of 5-HT in striatal LTP. In this regard, Ghiglieri and coworkers investigated the role of serotonin in corticostriatal LTP and synaptic depotentiation of SPNs, showing that 5-HT has no effect in physiological conditions. Indeed, bath application of the 5-HT_1A-1B_R agonist Eltoprazine, which leads to an increase in synaptic 5-HT levels, does not alter the normal induction of synaptic plasticity in adult rats [48]. However, a developmental effect has been revealed in a more recent paper, showing that, although 5-HT seems not to play a direct role in LTP expression, constitutionally low levels of 5-HT interfere with striatal LTP expression to a different extent in male and female mice [49].

## 2 DA AND 5-HT INTERACTION IN THE CORTICOSTRIATAL SYNAPTIC PLASTICITY IN PARKINSON’S DISEASE AND L-DOPA-INDUCED DYSKINESIA

Considering the multiple functions attributed to the serotonergic system, it is expected that any alteration in the 5-HT neurotransmission may potentially contribute to the modifications of DA-dependent corticostriatal plasticity, typical of the Parkinson's disease (PD) condition [50]. Even though administration of the DA precursor levodopa (L-DOPA) remains the gold standard treatment for PD patients, long-term treatments lead to the development of motor alterations, including involuntary movements known as L-DOPA-induced dyskinesias (LIDs), observed in the majority of patients [51, 52]. During the first decade of the 2000s, numerous studies on animal models clarified the pathophysiology of LIDs, providing the first evidence that striatal serotonergic terminals might be involved in the excess of DA release that follows the injection of its metabolic precursor [53-55]. Since then, the serotonergic system gained consideration as a potential target for PD and LIDs therapy [56].

**Fig. (3):**
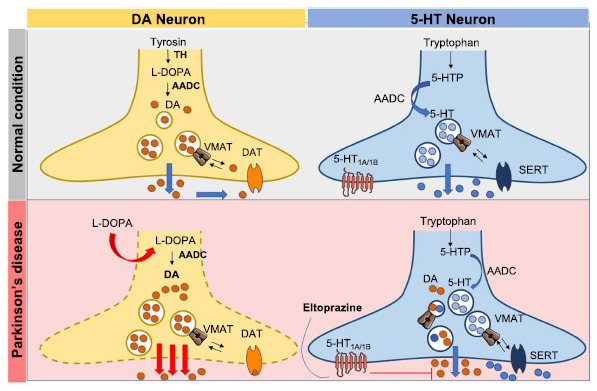
Synapses of the serotonergic and dopaminergic pathway in the normal condition and in the context of PD pathogenesis. Dopaminergic neurons that project from the substantia nigra to the striatum degenerate when Parkinson's disease progresses. Hence, under pathological conditions, serotonergic neurons originating in raphe nuclei can convert L-DOPA into dopamine, releasing it into the synaptic cleft *via* exocytosis. Unlike dopaminergic neurons, serotonergic neurons do not have the autoregulatory machinery to fine-tune the dopamine release. This determines an uncontrolled release of the neurotransmitter.

The first observations were based on the assumption that serotonergic neurons share with dopaminergic terminals the same enzymes necessary to convert L-DOPA into DA and release it, the aromatic amino acid decarboxylase (AADC) and the monoamine vesicular transporter (VMAT), respectively (Fig. **3**) [57]. Due to these features, serotonergic terminals hold the potential to compensate for the loss of nigrostriatal dopaminergic terminals. However, this neuronal population does not have a presynaptic expression of the DA active transporter (DAT) and, therefore, cannot operate any control on the release of DA itself. For these characteristics, 5-HT neurons can uptake L-DOPA and release DA in the extracellular space without ensuring its reuptake [58, 59]. The compensation of DA release is extremely efficient, resulting in structural plastic changes, as shown in animal models and human studies where excessive sprouting of serotonergic terminals is observed [55, 60, 61]. These changes further support the role of the serotonergic system in the development of LIDs by causing excessive synaptic peaks of DA, contributing to unphysiological oscillations of DA levels that follow the oral administration of the drug (Fig. **3**) [51, 62, 63]. As a consequence, a pulsatile stimulation of the striatal postsynaptic dopaminergic receptors induces critical changes in the D_1_R downstream signaling cascade [53, 56, 64, 65], lately associated with typical excitotoxicity and inflammatory processes [66, 67] associated with LIDs [68].

### 2.1 A New Mouse Model to Study Serotonin Contribution to the Striatal Function

**Fig. (4):**
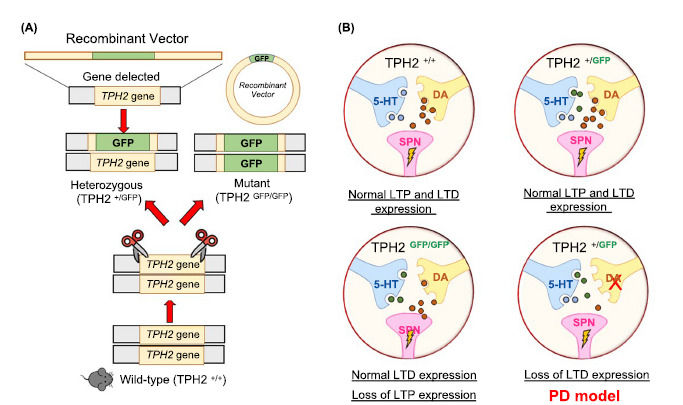
A new transgenic model to investigate the physiological roles of 5-HT. (**A**) Generation of the mutant TPH2^GFP/GFP^ mouse line and heterozygous TPH2^+/GFP^ in which the tryptophan hydroxylase 2 (TPH2) gene is replaced by the eGFP reporter in a way to highlight the serotonergic neurons. (**B**) Under physiological conditions, long-term depression (LTD) is typically expressed in all experimental groups. Instead, LTD is not expressed in the heterozygous group with dopaminergic denervation. In the normal condition, long-term potentiation (LTP) is absent only in TPH2^GFP/GFP^ mice.

A recent study further investigated the role of 5-HT in the organization of striatal functions. A novel experimental model, the tryptophan hydroxylase 2 (TPH2) knockin mouse, has been developed to analyze the role of the serotonergic system in regulating DA-dependent corticostriatal synaptic plasticity [49]. In this transgenic model, the TPH2 gene, essential for the biosynthesis of 5-HT in the brain, is constitutionally inactivated and replaced by the reporter green fluorescent protein (GFP) gene to detect 5-HT deficiency in the brain (Fig. **4A**) [16]. The study analyzed the contribution of serotonergic neurotransmission through the raphe-striatal innervation on the membrane and firing properties of SPNs and on the ability of striatal neurons to express synaptic plasticity. Recordings from SPNs in the dorsolateral STR of experimental groups with a partial (Heterozygous, Het, TPH2^+/GFP^) or a total lack of 5-HT content (knock-in, KI, TPH2^GFP/GFP^) compared with wild-type mice (Wt, TPH2^+/+^), showed similar discharge firing patterns, resting membrane potential, and membrane resistance among groups.

Moreover, LTD in the dorsolateral STR could be typically induced in all the experimental groups. However, even a mild perturbation of dopaminergic tone - not sufficient, per se, to block LTD induction - can worsen the effects of transgene. In fact, the ability to express LTD under partial depletion of 5-HT, observed in the TPH2^+/GFP^ mice, was lost if DA content was also reduced by a concurrent partial lesion of the nigrostriatal pathway, a condition that mimics early parkinsonism. SPNs of animals with an intact 5-HT content and a partial nigrostriatal lesion could still express LTD, as previously demonstrated in other models of early parkinsonism [69, 70], confirming that this form of plasticity requires the cooperation of both monoamines in the regulation of its induction (Fig. **4B**) [49].

Relevant to LTP, this form of plasticity was only expressed in control and partially depleted groups (Fig. **4B**). These findings support the idea that a DA/5-HT synergistic action is also needed for efficient synaptic potentiation [49].

### 2.2 5-HT_1A_/_1B_Rs Agonism Reduces the Induction of L-Dopa-induced Abnormal Voluntary Movements

Several studies demonstrated that the modulation of the serotonergic system can affect LIDs by showing that 5-HT_1_R agonists exert antidyskinetic effects in non-human primate and rodent models [71-73]. The concept that emerged from these studies is that 5-HT_1A_R agonists act on the 5-HT_1A_ inhibitory autoreceptors located in the terminals of the raphe neurons. The activation of these receptors is able to reduce DA release from serotonergic terminals caused by chronic L-DOPA, thus attenuating the pulsatile stimulation of the DA receptors (Fig. **3**) [56, 59].

It has been demonstrated that LIDs are associated with glutamate-mediated neuronal overactivity and changes in postsynaptic density subunit composition of NMDAR [74].

In light of these findings, the treatment with agonists acting on both 5-HT_1A_R and 5-HT_1B_R with therapeutic effect on LIDs (Fig. **3**) [53, 72, 75] could generate a synergistic effect on dyskinesia suppression [53, 56, 73]. Based on these findings, a study tested the hypothesis that 5-HT_1A_/_1B_R agonism could significantly decrease LIDs in experimental PD by renormalizing DA and glutamate receptors at postsynaptic density, thus recovering typical synaptic alterations observed in SPNs of parkinsonian animals [48]. Eltoprazine presents a similar affinity to 5-HT_1A_R (Ki = 40 nM) and 5-HT_1B_R (Ki = 52 nM) [76], which can be expressed both at pre-and postsynaptic levels in different neuronal populations, including serotonergic neurons, glutamatergic corticostriatal projections, and SPNs [77-80]. Presynaptic 5-HT_1A_R and 5-HT_1B_R exert inhibitory effects, regulating neurotransmitter release. In the 6-hydroxydopamine (6-OHDA)-lesioned rats, as a model of fully symptomatic PD, SPNs of parkinsonian animals chronically administered with L-DOPA could express a recovery of LTP. However, in about half of the animals, this form of plasticity was abnormally persistent even upon application of an appropriate stimulation that can exert synaptic depotentiation, a form of homeostatic plasticity that brings the synapse to baseline pre-LTP activity [11, 50, 74]. Parkinsonian animals obtained with this model also show abnormal involuntary movements (AIMs), a feature resembling human LIDs. In this study, Eltoprazine co-administered with L-DOPA can reduce LIDs by reducing the overactivation of D1R and excessive expression of specific NMDAR subunits at the postsynaptic density. In SPNs recorded from animals treated with the two drugs, different effects were observed in distinct subpopulations of striatal neurons, with a net increase in the number of SPNs showing a full recovery of synaptic depotentiation. The data indicate that besides the behavioral amelioration, Eltoprazine restores synaptic scaling.

## 3 DA AND 5-HT INTERACTION IN THE PREFRONTAL CORTEX

The PFC is a critical portion of the cerebral cortex that mediates executive functions and cognitive processes. The cognitive tasks controlled by PFC are sensitive to many neurochemicals, and different neurotransmitter systems present distinct roles in PFC complex functions. DA modulates PFC excitatory synaptic transmission and plasticity [81]. Dopaminergic fibers, mainly originating from the ventral tegmental area (VTA) [82], are widely distributed in the PFC and target layer 2 and layer 5 [83]. DA plays a role in the development and maturation of the prefrontal areas, modulating proliferation, migration, and differentiation processes [84-87]. Accordingly, interferences during critical developmental periods could contribute to cortical dysfunction and neuropsychiatric disorders [88]. Moreover, overstimulation or understimulation of D_1_/D_5_R hampers cognitive performances during PFC-sensitive behavioral tasks [89, 90].

The 5-HT system is one of the earliest to develop in the cortical areas during embryonic development [91, 92]. In the cortex, most postsynaptic 5-HT_1A_Rs are expressed in glutamatergic neurons and located in the soma, the initial segment [93, 94], and dendrites [95, 96]. This specific distribution of 5-HTR on pyramidal neurons is essential to control output signals from the cortex [97].

Cognitive functions regulated by PFC are susceptible to changes in dopaminergic and serotonergic transmission [98]. It is now well-established that the activity of cortical neurons is modulated by both DA and 5-HT [99, 100]. It has been demonstrated that both pyramidal [101-103] and non-pyramidal [104] neurons of this brain area receive inputs from dopaminergic fibers. These neuronal subtypes show D_1_R and D_2_R [105], with their respective mRNAs [106] localized in the cell bodies. There is a cooperation between serotonergic and dopaminergic systems within the PFC in shaping the direction of synaptic plasticity towards potentiation or depression that targets level 5 pyramidal neurons by optimizing the excitation and inhibition balance (E/I balance) [107].

Concerning the control of DA release, several studies demonstrated a functional interaction between 5-HT and DA systems in PFC, showing that 5-HT application (1-10 μmol/L) or agonists application (BAY x 3702, clozapine, olanzapine, risperidone) leads to an increase of extracellular DA in a dose-dependent manner, an effect likely mediated by 5-HT_1_R [108, 109]. These effects were abolished by WAY100635, a 5-HT_1A_R antagonist (Fig. **1**) [110].

In addition, Bortolozzi *et al.* demonstrated that in PFC, the activation of 5-HT_2A_R by DOI agonist application facilitated DA release [111]. This increase can be blocked after administration of the selective 5-HT_2A_R antagonist M100907 [112]. In contrast, an agonist (Ro600175) of 5-HT_2C_R induces a decrease in DA efflux in the PFC, while inverse agonists or antagonists (SB242,084) produce an increase in DA release (Fig. **1**) [113].

Patch-clamp recordings of deep-layer PFC neurons in brain slices allowed us to investigate the effects of bath application of DA or 5-HT on evoked firing. Previous data demonstrated the ability of DA or 5-HT to enhance the excitability of these neurons [87, 114, 115]. Indeed, the co-application of these monoamines produced a consistent increase in excitability and firing that does not occur when applied alone. Moreover, the sequential application of 5-HT (10 μM) and DA (20 μM) increased firing activity with greater magnitude than observed when DA was applied at the same time point in the absence of 5-HT pretreatment. Therefore, the excitability of PFC neurons depends on the complex interaction between 5-HT and DA. Interestingly, when cells were first primed with DA, applying 5-HT led to an overall increase in firing rates compared to the increase observed in the absence of DA pretreatment [116].

The presence of functional 5-HT_1A_Rs is essential to modulate excitatory synaptic plasticity through D_1_R or D_2_R, determining the direction of long-term plasticity toward LTP (D_1_R) or LTD (D_2_R). Meunier and coworkers showed that simultaneous activation of 5-HT_1A_R and D_1_R, using the NMDARs modulation, promotes potentiation of excitatory and inhibitory circuits and consequently does not modify the net E/I balance [107]. However, by favoring the LTD of excitatory synapses upon activation of D_2_R - which also requires activation of functional 5-HT_1A_R - it was possible to shift the E/I balance towards inhibition. The interactions between 5-HT_1A_R and D_1_R or D_2_R dictate the entity of the Ca^2+^ influx through NMDAR favoring LTP or LTD. Therefore, increased Ca^2+^ transients associated with 5-HT_1A_R and D_1_R activation were responsible for inducing LTP, whereas decreased influx following 5-HT_1A_R and D_2_R activation leads to LTD induction [107].

## 4 DA AND 5-HT INTERACTION IN THE PREFRONTAL CORTEX IN PATHOLOGICAL CONDITIONS

PD is characterized by motor dysfunctions often preceded by cognitive deficits, which involve deregulation of the medial prefrontal cortex (mPFC). Abnormal alterations in PFC activity have been observed in many neurological and neuropsychiatric disorders, suggesting an influence by the monoamines DA and 5-HT [117]. In the PFC, it is possible to increase the synaptic concentration of the neurotransmitters DA and 5-HT through treatment with amphetamine. The drug binds and reverses the transporters, blocking the reuptake and thus leading to increased levels of neurotransmitters in the synaptic cleft [118, 119]. Mair and collaborators showed the depressing action of amphetamine on excitatory synaptic transmission by the release of DA acting at D_1_/D_5_Rs [120]. Several studies have suggested that dysregulation between DA and 5-HT systems and abnormal neuronal activity could be a common cause of different pathologies, including PD [56, 121]. These disorders are usually treated with drugs that target the DA and/or 5-HT systems [122-124]. Relative to normal controls, individuals with PD present cognitive deficits on prefrontal tasks and decreased activation of prefrontal circuits during self-generated motor movements in a task [125-127]. Furthermore, an increase in 5-HT_2A_R activation in the PFC could hypothetically lead to downstream changes in the corticostriatal and cortico-brainstem projections regulating DA release in the ventral STR in patients with PD [128, 129]. Therefore, blocking 5-HT_2A_R with the selective 5HT_2A_R antagonist Pimavanserin or with the non-selective 5HT_2A_R antagonist Quetiapine and Clozapine would ameliorate the 5-HT-DA imbalance, thus contrasting psychotic symptoms of PD [130].

Furthermore, in 2016, Matheus and collaborators extended the study on the functional alteration of the mPFC in parkinsonian rats obtained with a partial lesion of the nigrostriatal dopaminergic pathway through bilateral injection of 6-OHDA into the dorsolateral STR [131]. In particular, electrophysiological recordings showed that synaptic plasticity is altered in the mPFC in the early premotor stages of experimental PD with memory impairment. The authors observed an impairment of mPFC-LTP 21 days after the 6-OHDA lesion, with decreased LTP amplitude after the induction. At the same time point after 6-OHDA injection, they observed LTP impairment and a reduction in 5-HT levels in PFC. [132]. This finding was a further demonstration in support of a functional interaction between DA and 5-HT neurotransmitters in the regulation of PFC-related functions. The activation of other 5-HTR can modulate L-DOPA-stimulated DA release. In this regard, 5-HT_4_R stimulation can modulate L-DOPA-stimulated DA release. In this regard, their stimulation can enhance L-DOPA-induced DA release in the PFC [132].

## 5 DA AND 5-HT INTERACTION IN THE HIPPOCAMPUS

The HPC is a complex brain structure that represents an extension of the temporal part of the cerebral cortex. It plays an essential role in learning and memory and is responsible for forming, updating, and retrieving memories [133, 134]. To date, very few studies have clarified the mechanisms by which 5-HT-DA interaction regulates learning and memory, even if the available scientific data show a reciprocal modulatory action between these two neurotransmitter systems. The use of neurotransmitter agonists and antagonists allows us to study their activity and interaction by identifying an association between a 5-HT/DA imbalance and cytoarchitecture changes that underlie learning and memory impairment (Fig. **1**) [135].

### 5.1 General Features of Hippocampal Synaptic Plasticity

Hippocampal-dependent learning and memory depend on short- and long-lasting synaptic modifications, mostly occurring at dendritic spines. In detail, two forms of synaptic plasticity, LTP and LTD, have been extensively investigated in the HPC to unravel the molecular and cellular basis of learning and memory [136, 137]. Depending on the magnitude of afferent stimulation, it can direct plasticity at CA3-CA1 hippocampal synapses toward LTP or LTD. A low-frequency stimulation (600 pulses at 1 Hz) was able to lead to LTD expression; conversely, the high-frequency stimulation (2 trains of 100 pulses at 100 Hz) facilitates the expression of LTP, and both inductions depend on the activation of NMDAR and metabotropic mGluR [138]. The influx of Ca^2+^ through NMDAR triggers the subsequent changes in the expression of another class of glutamatergic receptors, the AMPAR that constitute the primary component of the basal excitatory postsynaptic potential (EPSP). The degree of NMDAR activation depends in part on extracellular Mg^2+^ levels and the simultaneous presence of other agonists of this receptor, glycine, and D-serine [138].

### 5.2 Roles of DA in Hippocampal Synaptic Plasticity

DA plays an essential role in facilitating the LTP induction in the CA1 region through activation of the D_1_/D_5_R family. This ability is lost after the application of the selective D_1_R antagonist SCH23390. Indeed, pretreatment with an intra-cerebro-ventricular injection of SCH23390 (15 µg in 5 µl water vehicle) 50 minutes before the introduction of the animal into the novel environment for 5 minutes blocks LTP induction [139]. Instead, injecting the D_1_/D_5_ agonist SKF38393 at a dosage that did not affect baseline transmission facilitated LTP induction. Therefore, in the intact HPC, D_1_/D_5_R activation is necessary to facilitate novelty-induced LTP, which is sufficient to mediate it [140]. These results demonstrated how novelty exposure can control learning by activating mesolimbic dopaminergic neurons [140, 141]. In addition, a recent study suggests a role for DA in the long-term regulation of inhibition in the CA3 area, which is critical for memory consolidation [142].

### 5.3 Roles of 5-HT and 5-HT/DA Interaction in Hippocampal Synaptic Plasticity

5-HT is released by fibers projecting diffusely from the median raphe nucleus into all the hippocampal areas [143]. All 5-HTR (5-HT_1_R to 5-HT_7_R) are expressed in the HPC [144, 145]. In this brain area, 5-HT directly inhibits pyramidal neurons *via* 5-HT1_A_R and indirectly facilitates GABA release by interneurons through 5-HT_3_R activation [146]. The 5-HT1_A_R activation, through its agonist 8-hydroxy-2-(di-n-propylamino)-tetralin (8-OHDPAT), has effects on DA neurotransmission, reducing 5-HT and increasing DA levels in the HPC of rats (Fig. **1**) [147].

Another interesting result was observed using SB399885, a selective 5-HT_6_Rs antagonist, at 3 and 10 mg/kg dosages, which increased DA release in the HPC (Fig. **1**). Of particular interest was the ability of SB399885, 3 mg/kg, to increase significantly the action of an antipsychotic drug, haloperidol, a D_2_R antagonist, increasing DA release in the HPC [148]. 5-HT was able to increase cellular excitability through several mechanisms, such as inhibition of K^+^ channels, enhancement of NMDAR activation [149], and facilitation of LTP expression in CA1 [150-152]. Bliss and coworkers showed a blockade of hippocampal LTP following exogenous 5-HT bath application in hippocampal slices [153].

In contrast to the application of exogenous 5-HT, the release of endogenous 5-HT does not impair the cellular mechanisms responsible for LTP induction [153], demonstrating that 5-HT is not detrimental to learning and memory. Furthermore, the ability of endogenous 5-HT to facilitate LTP may underlie the *in vivo* beneficial effects on cognitive performances associated with increased 5-HT tone [153]. In CA3, the 5-HT exerts mainly inhibitory action and prevents LTD or LTP at mossy fiber-CA3 synapses [154], highlighting the cell-specific nature of serotonergic modulation in this area. Interestingly, in rat hippocampal slices, besides inhibiting the induction of LTP in the CA1 [155-157], the 5-HT application could also block LTP in CA3 regions [153, 158]. In fact, LTP can be facilitated by the blockade of 5-HT_3_R *via* the selective antagonist ondansetron [156].

The 5-HT_4_R are also involved in the hippocampal long-term synaptic plasticity. Activation of the 5-HT_4_R has been shown to prevent hippocampal LTD [151, 154]. However, the effect of these receptors on the LTP is more complex and can change according to the expression of the receptor isoforms [159]. Numerous studies have shown that stimulation of 5-HT_4_R promotes LTP in the CA1 region [151] and the dentate gyrus [154, 160]. Conversely, activation of the 5-HT_4_R in the CA3 region leads to the suppression of LTP [154]. This evidence suggests that both 5-HT and DA are essential in cognitive activity. However, all the mechanisms of interaction between the two monoamines at the hippocampal level are far from being fully clarified [161].

The interplay between DA and 5-HT seems to regulate cognitive functions underpinning decision-making [162]. Evidence suggests that the antagonistic effects of DA and 5-HT can shape neural activity during reward-driven learning [163-165]. In detail, while DA is responsible for inducing LTP in reward-guided navigation [143, 166], 5-HT favors LTD for some receptor-specific hippocampal areas [167] but can also induce hippocampal LTP. Low doses of 3,4-methylenedioxymethamphetamine (MDMA), commonly known as Ecstasy, have been shown to lead to increased release of various neurotransmitters, including endogenous 5-HT. The induction of LTP was abolished following the use of citalopram, an SSRI that prevented MDMA entry into serotonergic terminals and subsequent 5-HT release [153].

## 6 DA AND 5-HT INTERACTION IN HIPPOCAMPAL DYSFUNCTIONS

The HPC is implicated in the cognitive dysfunction typical of PD [168]. As an important neurotransmitter involved in the motivation and stimulus-reward learning process, DA could also modify the hippocampal synaptic plasticity, affecting the intrinsic properties of the neuronal cells, such as membrane excitability and the probability of neurotransmitter release [169]. Similar to DA, the alteration of the serotonergic system is also involved in the memory impairment found in Parkinsonian subjects [170].

The facilitation of hippocampal memory extinction and its impairment have been studied in PD mice obtained *via* 1-methyl-4-phenyl-1,2,3,6-tetrahydropyridine (MPTP). Cognitive alterations can be reverted by 5-HT_4_R agonists Prucalopride and Velusetrag through cAMP/CREB pathway stimulation in the dentate gyrus (DG). These results suggested that 5-HT_4_R agonists could be used as drugs to treat cognitive deficits in PD [171]. In this regard, many pharmacological studies have focused on the role of 5-HTR in learning and memory processes [172]. In addition, the increase of alpha-synuclein (a-syn) levels, the major component of the typical aggregates found in PD, is associated with a reduction in 5-HT levels in selective hippocampal sub-regions (DG/CA3) and intense growth of SERT^+^ fibers projecting into the DG, indicating the presence of dystrophic hippocampal SERT^+^ fibers in this PD model [173].

Furthermore, both a reduction of 5-HT hippocampal levels and a decrease of SERT binding were observed in PD models, describing a dysfunctional serotonergic system [174]. Interestingly, the 5-HT_1A_R levels in the HPC of the a-syn BAC rats, a genetic model of PD in which a-syn is selectively expressed in a given neuronal population, did not change. In contrast, the 5-HT_1B_R levels were reduced. This presynaptically localized receptor plays an essential function in the control of neurotransmitters. Therefore, the loss of serotonergic terminals within the target field of the HPC may reflect an early stage of a-synucleinopathy, confirming the hypothesis that neuronal loss in PD is preceded by synaptic loss and axonal degeneration [175, 176].

Despite the importance of these two neurotransmitters in the impairment of learning and memory in PD, few studies have investigated their synergistic interaction at the hippocampal level, and the mechanisms underlying their dysregulated interactions are still unclear.

## CONCLUSION

Taken together, all of these studies provided an outstanding contribution to the overview of the interaction between DA and 5-HT in STR, HPC, and PFC regions, describing how activation or inhibition of a precise class of receptors leads to changes in these neurotransmitter expression levels, affecting synaptic plasticity and behavior.

The roles of these two monoamines and their interaction in regulating synaptic plasticity underlying learning and memory functions have been extensively described in the present review.

It can be inferred that the two neurotransmitters interact in different ways with a mutual modulatory action and distinct physiological characteristics depending on the brain region involved and the conditions considered, with a particular focus on PD.

The interaction between DA/5-HT has been a subject of debate for several decades, and the leading concept is that intact crosstalk is needed to ensure learning and memory mediated by DA in the STR and PFC. In addition, there seems to be a mutual modulation between the two neurotransmitter systems, also in the HPC, a region that is not directly involved in the regulation of voluntary movements.

Future studies, providing further insights into the mechanisms and clinical implications of 5-HT/DA interaction, would be of pivotal importance to enhance the understanding of motor learning and develop therapeutic strategies for treating motor and cognitive alterations in PD patients.

## LIST OF ABBREVIATIONS

A-SYN: Alpha-synuclein
AADC: Aromatic Amino Acid Decarboxylase
AIMs: Abnormal Involuntary Movements
AMPAR: α-amino-3-hydroxy-5-methyl-4-isoxazole Propionic Acid Receptors
D_1_R: D_1_ Dopamine Receptors
D_2_R: D_2_ Dopamine Receptors
DA: Dopamine
DAT: Dopamine Active Transporter
DS: Dorsal Striatum
eCB: Endocannabinoids
GABA: Gamma-aminobutyric Acid Striatal
GFP: Green Fluorescent Protein
5-HT: Serotonin
5-HTR: 5-HT Receptors
6-OHDA: 6-hydroxydopamine
8-OHDPAT: 8-hydroxy-2-[di-n-propylamino]-tetralin
HFS: High-frequency Stimulation
HPC: Hippocampus
IHC: Immunohistochemistry
K^+^: Potassium Ion
LIDs: Levodopa-induced Dyskinesias
LTD: Long Term Depression
LTP: Long Term Potentiation
Mg^2+^: Magnesium Ions
mGluR: Metabotropic Glutamate Receptors
mPFC: Medial Prefrontal Cortex
MPTP: 1-methyl-4-phenyl-1,2,3,6-tetrahydropyridine
mRNA: Messenger RNAs
NMDAR: N-methyl-D-aspartate Channel Receptor
PD: Parkinson's Disease
PFC: Prefrontal Cortex
SERT: Serotonin Transporter
SN: Substantia Nigra
SPNs: Spiny Projection Neurons
SSRI: Selective Serotonin Reuptake Inhibitors
STR: Striatum
TPH2: Tryptophan Hydroxylase 2
VMAT: Monoamine Vesicular Transporter
VTA: Ventral Tegmental Area
